# Impact of Silver Nanoparticles on Haemolysis, Platelet Function and Coagulation

**DOI:** 10.5772/59346

**Published:** 2014-01-01

**Authors:** Julie Laloy, Valentine Minet, Lutfiye Alpan, François Mullier, Sonja Beken, Olivier Toussaint, Stéphane Lucas, Jean-Michel Dogné

**Affiliations:** 1 Department of Pharmacy, Namur Nanosafety Center (NNC), NAmur Research Institute for Life Sciences (NARILIS), NAmur MEdicine & Drug Innovation Center (NAMEDIC), Namur Thrombosis and Hemostasis Center (NTHC), University of Namur, Namur, Belgium; 2 Hematology Department, CHU Dinant Godinne - UCL Namur, Belgium; 3 Division Evaluators, DG PRE Authorisation, Federal Agency for Medicines and Health Products (FAMHP), Brussels, Belgium; 4 Laboratory of Cellular Biochemistry and Biology, NNC, NARILIS, University of Namur, Namur, Belgium; 5 Research Centre for the Physics of Matter and Radiation, NNC, NARILIS, University of Namur, Namur, Belgium

**Keywords:** Silver Nanoparticles, Red Blood Cells, Platelets, Coagulation

## Abstract

Silver nanoparticles (Ag NPs) are increasingly used in biomedical applications because of their large antimicrobial spectrum. Data in the literature on the ability of Ag NPs to perform their desired function without eliciting undesirable effects on blood elements are very limited and contradictory. We studied the impact of Ag NPs on erythrocyte integrity, platelet function and blood coagulation. Erythrocyte integrity was assessed by spectrophotometric measurement of haemoglobin release. Platelet adhesion and aggregation was determined by light transmission aggregometry and scanning electron microscopy. The calibrated thrombin generation test was used to study the impact on coagulation cascade. We demonstrated that Ag NPs induced haemolysis. They also increase platelet adhesion without having any impact on platelet aggregation. Finally, they also had procoagulant potential. Bringing all data from these tests together, the no observed effect concentration is 5 μg/mL.

## 1. Introduction

Silver has been used in medicine for centuries on account of its antimicrobial properties [1,2]. Because of their large surface area-to-volume ratios, silver nanoparticles (Ag NPs) offer a large antimicrobial spectrum and greater efficacy against bacteria than common antibiotics [3-5]. Ag NPs provide new opportunities for use in both consumer and biomedical applications, making them the most commercialised NPs [6]. For example, they are integrated into garments in order to neutralize odourforming bacteria [7], they coat various food contact materials (refrigerator surfaces, storage bags…) on purpose to inhibit microorganism growth and to preserve food longer [8]. They are also used in disinfectants, deodorants, water purificants, antimicrobial sprays, etc. [9]. In the medical field, Ag NPs coat cardiovascular and neurosurgical catheters, orthopaedic and cardiovascular implants, surgical instruments, wound and burn dressings, and bone substitute biomaterials [1, 3, 10, 11]. Other biomedical applications of Ag NPs include drug delivery systems and targeting [12]. Moreover, Ag NPs have demonstrated anti-inflammatory effects, improving the outcome of the healing process [13, 14].

In some biomedical applications, Ag NPs are directly in contact with blood. Because of their reduced particle size and increased surface area Ag NPs may also enter the body and translocate into systemic blood flow after inhalation, ingestion, dermal contact or systemic administration [9, 15-17]. Ag NPs, after inhalation, oral administration or subcutaneous injection in rats, may reach the blood stream and be distributed to organs including liver, kidney and brain [15-19]. Information on the potential impact of Ag NPs on haemolysis, platelet activity and coagulation associated with human exposure is however very limited [3, 10]. An important haemolysis (destruction of red blood cells (RBC)) may result in pathological conditions such as anaemia and renal failure [20]. Moreover, a homeostatic imbalance in platelet function (primary haemostasis) or in the coagulation system (secondary haemostasis) can lead to thrombotic or haemorrhagic disorders. The plasma coagulation cascade, responsible for blood clotting, consists in a series of sequential enzymatic reactions performed by activated coagulation factors. It can be triggered through two distinct pathways: the cell-mediated tissue factor (TF) pathway and the surface-mediated contact pathway (formerly known as extrinsic and intrinsic pathways, respectively). Both pathways converge to a common pathway resulting in active thrombin generation and fibrin clot formation. The majority of these proteolytic reactions occur on anionic phospholipid membrane surfaces from activated platelets or endothelial cells *in vivo* and require calcium as a cofactor [21]. There is therefore a plausible possibility that some nanomaterials interfere with some of these reactions, as recently demonstrated [22]. In the present study, we focused on the effects of Ag NPs on three parameters of blood: (i) haemolysis, (ii) primary haemostasis and (iii) secondary haemostasis.

It is proposed that the no observed effect concentration (NOEC) identified for a particular NP in the most sensitive *in vitro* assay of the haemocompatibility testing battery could be used to guide the selection of the human free plasma concentration that could be considered safe from a haemocompatibility perspective for a first-inhuman exposure, and to identify appropriate safety margins (i.e., the ratio between the NOEC and the human free plasma concentration at the clinical starting dose). In addition, the lowest effect concentration (LOEC) for a particular NP could similarly be extrapolated to estimate the human plasma concentration where haematotoxicity would be expected to occur (toxic concentration). Both NOEC and LOEC will be determined.

## 2. Methods

### 2.1 Nanoparticle characterization

Ag NPs were obtained from the European project NanoValid. They were suspended in water at a stock concentration of 40 mg/mL, stabilized by polyvinyl pyrolidone (PVP) and stored at 4°C. Ag NPs were diluted in tyrode buffer (10 mM Hepes, 6 mM NaHCO_3_, 137 mM NaCl, 2.7 mM KCl, 5 mM glucose, 1 mM MgCl_2_ and 0.8 mM NaH_2_PO_4_, diluted in ultrapure water (milliQ 18.2 MW.cm), pH 7.4).

A proper characterization of Ag NPs was performed as previously reported [23]. Briefly, the bulk composition was determined by energy dispersive X-ray (EDX). The surface composition was analysed with an X-ray photoelectron spectroscopy (XPS) system. A droplet of 10 μL of the Ag NPs dispersion was left to dry on a gold-covered silicon wafer. The particle size distribution was measured with a DC24000 system Disc Centrifuge (CPS Instruments Inc.). A certified PVC microparticle calibration standard (226 nm), provided by the instrument supplier, was used to calibrate all measurements. The volume injected was 0.02 mL for each experiment.

### 2.2 Preparation of human platelet-rich plasma (PRP), platelet-poor plasma (PPP) and washed red blood cells (RBC) suspension and normal pooled plasma (NPP)

PRP, PPP, whole blood, washed RBC suspension and NPP were prepared with blood from healthy volunteers who were free from any medication for at least two weeks. Blood was collected by venepuncture into tubes containing buffered sodium citrate (109 mM, nine parts blood to one part of sodium citrate solution) (BD Vacutainer®). The study protocol was in accordance with the Declaration of Helsinki and was approved by the Medical Ethical Committee of the CHU Dinant-Godinne UCL Namur (Yvoir, Belgium).

Platelet-rich plasma (PRP) was carefully prepared by centrifugation at 200 *g* at room temperature for 10 min. The platelet count was adjusted to 300,000 platelets/μL and PRP were used immediately after preparation.

Platelet-poor plasma (PPP) was subsequently obtained by centrifugation at 2,000 *g* of the pellet at room temperature for 10 min. It was frozen at −80 °C immediately after centrifugation. It was defrosted at 37 °C just before use.

To prepare a suspension of washed RBC in PBS, whole blood is centrifuged at 3,000 *g* over 5 min. The PPP is removed and used for interference assays. RBC are washed with physiological phosphate buffered saline (PBS, 6.7 mM phosphate, pH=7.4) three times with intermediate centrifugation of 3,000 *g* over 5 min. They are then resuspended in PBS with the same volume as PBS removed.

For NPP, a total of 40 healthy individuals were included in the study. The exclusion criteria were thrombotic and/or haemorrhagic events, antiplatelet and/or anticoagulant medication, pregnancy and uptake of drugs potentially affecting the platelet and/or coagulation factor functions during the two weeks prior to the blood drawn. A written informed consent was obtained from each donor. The study population displayed the following characteristics: 24 females and 16 males aged from 19 to 48 years (mean age = 25 years) with body mass index (BMI) ranging from 16.8 to 34.6 kg/m^2^ (mean BMI = 22.3 kg/m^2^). After collection of blood, the PPP was obtained from the supernatant fraction of the blood tubes after a double centrifugation for 15 minutes at 2,000 *g* at room temperature. It was immediately frozen at −80°C after centrifugation. The NPP samples were thawed and kept at 37 °C just before use.

### 2.3 Haemolysis assays

Haemolysis assays were performed on the blood of one healthy donor: 15 μL of nanoparticles (suspended in tyrode), tyrode (negative control) or triton X-100 (positive control) are added to 285 μL of whole blood (protocol 1) or washed RBC (protocol 2). The suspension is incubated at room temperature on a shaking plate during 1 h, 4 h and 24 h. After the incubation time, the suspension is centrifuged at 10 000 g over 5 min. Supernatant is read in a 96-well plate using a microplate scanning spectrophotometer XMark (Biorad, USA) at 550 nm. The % haemolysis was calculated as: H (%) = (OD550nm sample –OD_550nm_ tyrode)/(OD_550nm_ Triton X-100 1% – OD_550nm_ tyrode)*100. For each term of the equation, the corresponding interference was subtracted. The interference corresponds to the same conditions except that the solution does not contain RBC. Positive and negative controls induced 100% and 0% of lysis, respectively. The results were expressed as means ± SD (n = 3).

### 2.4 Platelet functional assays

#### 2.4.1 Light transmission aggregometry (LTA)

The impact of Ag NPs on spontaneous or induced platelet aggregation was studied using the chronometric aggregometer type 490–2D as previously reported [24]. The reaction mixture for spontaneous aggregation tests contained 285 μL of PRP at 300,000 platelets/μL and 15 μL of NPs with final concentrations from 1 to 25 μg/mL. The reaction mixture for induced aggregation tests contained 255, 255 or 280 μL of PRP at 300,000 platelets/μL, with respectively 30 μL of ADP (final concentration: 20 μM, Bio/Data corporation, USA), 30 μL of collagen (final concentration: 190 μg/mL, calf skin, Bio/Data corporation, USA) or 6 μL of arachidonic acid (AA, final concentration: 600 μM, Calbiochem, Germany) and 15 μl of NPs at final concentration from 1 to 25 μg/mL. Inducers alone were also used before any experiment to check platelet reactivity. PPP was used as a reference. Data were collected with the chronolog two-channel recorders at 405 nm connected to a computer.

#### 2.4.2 Scanning electron microscopy (FEG-SEM)

Preparation of samples was similar to LTA. The samples of PRP (500 μL) were deposited on a glass slice coated with poly-L-lysine; 500 μL of glutaraldehyde (final concentration: 2.5%) in cacodylate buffer (Na(CH_3_)_2_.AsO_2_.3H_2_O in distilled water at pH 7.4, final concentration: 0.1 M) was added over 90 minutes. Samples were then centrifuged at 2,000 *g* for platelet adhesion onto the slice. The glutaraldehyde solution was removed and 400 μL of 0.2 M cacodylate buffer was added. The next step is the dehydration of the sample with successive baths of alcohol from 30° to 100°. A critical point drying was performed with a specific apparatus: the Balzers Critical Point Dryer (CPD) 030 (BAL-TEC GmbH®, Germany). Afterwards, a thin layer of platinum (20 nm) was deposited under argon atmosphere on the sample with the metallizer Balzers union (BAL-TEC GmbH®, Germany). The samples were observed with FEG-SEM (Jeol, Japan; resolution of 0.6 nm at 20 keV) [22].

### 2.5 Coagulation: Calibrated thrombin generation test (cTGT)

The impact of Ag NPs on coagulation was studied using the calibrated thrombin generation test (cTGT) as previously reported [22]. For each experiment, a fresh mixture of fluorogenic substrate/calcium chloride buffered solution was prepared as follows: 2.6 mL of Fluo Buffer® (Thrombinoscope BV, The Netherlands) were mixed with 65 μL of Fluo substrate® (100 mM in DMSO, Thrombinoscope BV, The Netherlands). PPP-Reagent, PPP-Reagent LOW, MP-Reagent and Thrombin Calibrator (Thrombinoscope BV, The Netherlands) are four inducers, giving final assay concentrations of 5 pM tissue factor (TF) with 4 μM phospholipids (PL) and 16.7 mM CaCl2; 1 pM TF with 4 μM PL and 16.7 mM CaCl_2_; 4 μM PL and 16.7 mM CaCl_2_; and 620 nM α2-macroglobulin-thrombin complex, respectively. They are reconstituted with 1 mL distilled water according to the instructions provided by the manufacturer. A calibration curve was simultaneously performed using the thrombin calibrator. The acquired data were automatically processed by the software, which provided thrombin activity curves and lag time (minutes), C_max_ (nM) and endogenous thrombin potential (ETP, nM*minutes) parameters. The NP suspensions were tested at final concentrations from 5 to 500 μg/mL. Statistical analyses were conducted with an unpaired t-test using the GraphPad Prism software (GraphPad software, v 5.01, USA).

### 2.6 Interferences of NPs with the detection methods used

#### 2.6.1 NP interferences with methods using light absorbance detection

Haemolysis assay and LTA use light absorbance detection. In order to study light interference of NPs, 20 μL of Ag NP suspensions and 180 μL of suspension agent were mixed in a 96-well polystyrene plate (Greiner Bio One, Belgium). The light absorbance was measured from 400 to 800 nm using a microplate absorbance spectrophotometer XMark (Biorad, USA). For haemolysis assay, the interference was avoided by the subtraction of the OD_550nm_ of NPs suspended in the vehicle from the measured OD_550nm_ at the same concentration. For LTA, the NP concentration requiring an increase of 0.1 unit of the OD at 405 nm (Δ0.1 OD_405nm_) was calculated from the plot of the OD_405nm_ against the NP concentration by a linear regression curve using the GraphPad Prism® software (GraphPad Prism software, v 5.01, USA). The lower this parameter, the more NP suspensions absorb light.

#### 2.6.2 NP interferences with the cTGT method

The autofluorescence of the NP suspensions was measured at 390/460 nm using the microplate fluorometer Fluoroskan Ascent® FL (Thermo Labsystems, The Netherlands). Furthermore, the quenching of 7-amino-4-methyl coumarin acetic acid (AMC) by the NP suspensions was also measured.

## 3. Results

### 3.1 Nanoparticle characterization

Ag NPS have a spheroid shape.

A bimodal distribution was observed with primary particles around 16 nm and agglomerates around 71 nm (Table 1). The NP surface composition was not similar to their bulk composition with a lower content of oxygen and the presence of nitrogen. The elements other than Ag are attributed to traces of the suspension solution.

**Table 1.** Summary of Ag NPs characterization

Parameters	Measurement
Hydrodynamic diameter (CLS, [nm])	16 (primary particles), 71 (agglomerates)
Elemental composition	Ag O C N Na
Surface composition (XPS, [% at])*	5 23.8 61 9.2 1
Bulk composition (SEM-EDX, [% at])*	4.71 34.22 60.78–0.29

* Ag NPs are dispersed in water and stabilized with PVP

### 3.2 Haemolysis

We performed the absorbance spectrum of the supernatant of RBC suspension 0.5% (v/v) incubated with Triton X-100 1% (v/v) (data not shown). We selected the wavelength of 550 nm to perform our assay. Note that OD interference has been observed with Ag NPs due to their absorbance at 550 nm. This interference was avoided by the subtraction of the OD_550 nm_ of NPs suspended in the vehicle from the measured OD_550 nm_ at the same concentration. Negative control (tyrode) and positive control (triton X-100 1%) induced 0% and 100% respectively of haemolysis.

**Figure 1.**
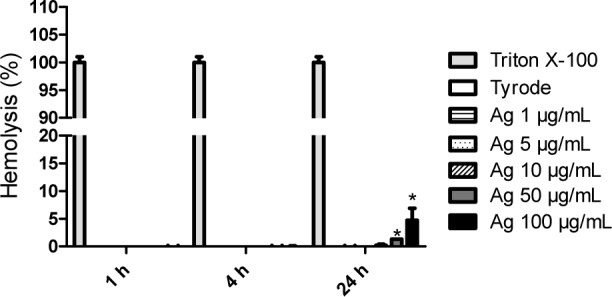
Human RBC lysis (%) induced by Ag NPs in washed RBC after 1 h, 4 h and 24 h at concentrations ranging from 1 to 100 μg/mL (protocol 2). Triton X-100 1% and tyrode (v/v) are respectively used as positive control and negative control. Mean (%) ± SD, n = 3. Statistical analysis was performed with GraphPad Prism software (unpaired t-test). Statistical significance between control and Ag NPs samples: * indicates p<0.05.

Ag NPs at 1 μg/mL, 10 μg/mL and 100 μg/mL induced no haemolysis in whole blood (data not shown) after 1 h, 4 h or 24 h (data not shown). With washed RBC, no haemolysis was observed, except for the concentration of 100 μg/mL after 24 h of incubation. A statistically different percentage of haemolysis (4.7%) is obtained (Figure 1).

### 3.3 Platelet function

To evaluate the suitability of LTA for Ag NPs, the light absorbance properties of these NPs was determined. Ag NP suspensions absorb light between 400 and 800 nm.

According to the detection sensitivity of LTA, the NP concentration required to obtain a 0.1 unit increase in the OD at 620 nm (Δ0.1 OD_620 nm_) was measured; significant light absorbance was observed from a concentration higher than 10 μg/mL Ag NPs.

**Figure 2.**
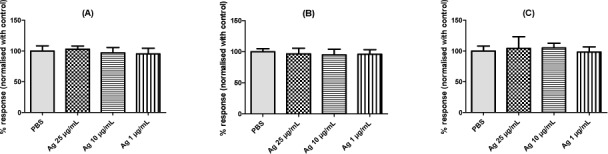
Platelet aggregation induced by ADP (A), collagen (B) or AA (C) at different concentrations of Ag NPs. PBS is used as a negative control. Results are expressed as % of response (Mean ± SD, n = 6, unpaired t-test).

The potential impact of Ag NPs on platelet activity was investigated at the concentration without interference in tested conditions (< 25 μg/mL). The cut-off was arbitrarily defined at 5% reduction of the aggregation by agonist in control conditions. At the investigated concentrations avoiding interferences, Ag NPs did not significantly affect platelet aggregation, regardless of the inducer used (ADP, collagen or AA) (Figure 2). In the absence of agonists, Ag NPs did not induce spontaneous platelet aggregation (data not shown).

**Figure 3.**
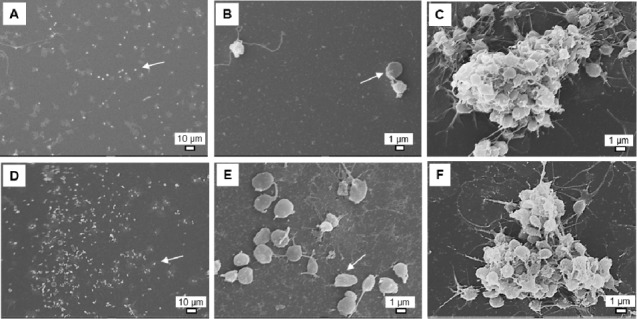
SEM pictures of platelet adhesion without (A -C) or with Ag NPs at a final concentration of 50 μg/mL (D -F). Platelet aggregation was induced by AA (C, F). Scale bare = 10 μm (A, D) or 1 μm (B, C, E, F). Arrows indicate adherent platelets.

In FEG-SEM, a reduction of platelet adhesion is observed with Ag NPs at 50 μg/mL (Figure 3D and E). FEG-SEM does not allow conclusion of a potential effect of Ag NPs on the platelet activation and aggregation (Figure 3).

### 3.4 Coagulation: Calibrated thrombin generation test (cTGT)

The investigations of the two potential interferences of Ag NPs with cTGT indicated that Ag NPs were not autofluorescent at wavelengths of 390/460 nm, but they were able to reduce the fluorescent signal of the AMC fluorophore. This interference can be excluded by performing a specific calibration curve for each concentration tested [22].

**Figure 4.**
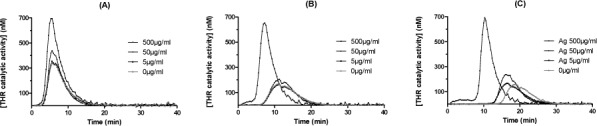
Representative thrombin activity profiles induced by TF (A), contact (C) or both pathways (B) in the presence of Ag NPs. Data shown are the means of three independent experiments.

**Figure 5.**
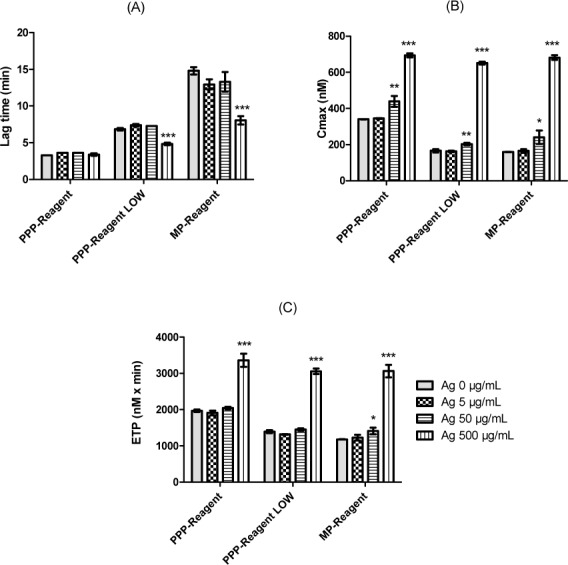
Control parameters (lag time (A), C_max_ (B) and ETP (C)) of thrombin activity profiles induced by TF, contact or both pathways of negative control and different concentrations of Ag NPs. Statistical analysis was performed with GraphPad Prism software (unpaired t-test). Statistical significance between control and Ag NPs samples: * indicates p<0.05; ** indicates p<0.01; *** indicates p<0.001.

Figure 4 shows representative active thrombin profiles after activation by the use of three specific inducers in the presence of Ag NPs. Figure 5 represents the control parameters. Firstly, the investigation of the TF pathway showed that only a high concentration of Ag NPs (500 μg/mL) increases the concentration of generated thrombin (C_max_) (Figure 4A and Figure 5B). Secondly, the active thrombin was generated through the TF pathway, but with a lower concentration of TF in order to allow the thrombin-positive feedback loop activation on the contact pathway (Figure 4B). In these specific activation conditions, it appeared that high concentration decreased lag time and increased C_max_ (Figure 5A and B).

When the contact pathway was triggered, Ag NPs showed a procoagulant activity with a decrease of lag time and an increase of C_max_ (Figure 4C). Based on these results, it appears that Ag NPs have a procoagulant activity by reducing lag time and by increasing Cmax when the contact pathway (4 μM PL) is preferentially stimulated.

### 3.5 No/low observed effect concentration

NOEC and LOEC were determined for blood parameters studied. For red blood cells, the NOEC and LOEC were 50 μg/mL and 100 μg/mL, respectively. The NOEC and LOEC were 5 μg/mL and 50 μg/mL for coagulation, respectively. For platelet function, due to the technical limitations of LTA, the NOEC is 25 μg/mL and no LOEC could be determined. The effects on platelet adhesion observed in FEG-SEM are a qualitative result allowing no NOEC and no LOEC.

## 4. Discussion

The increasing use of Ag NPs leads to the need of toxicological evaluation to insure patient safety [25]. There is limited information on the biological effects of Ag NPs on human blood cells and the potential risk of inducing thrombosis [11]. Moreover, some published studies do not provide information on interferences of the assays. In addition, results are sometimes contradictory [26-29]. Physicochemical properties of NPs are of major importance in toxicology and may be affected by the dispersing media [30, 31]. Therefore, results of blood assays can differ according to the media used. We suggested that the NP suspensions should be prepared in the most physiological media suitable for blood components (tyrode buffer), avoiding sonication [22, 25]. As their unique size and composition affect their functionality, complete physicochemical description of nanoparticles was performed, allowing the comparison of results between scientific studies [25].

The determination of haemolysis is based on haemoglobin absorbance at 550 nm, with subtraction of the interference of Ag NPs. A significant difference was observed in washed RBC with Ag NPs at 50 and 100 μg/mL after 24 h of incubation in comparison with controls. A lower haemolytic effect was observed in whole blood than in washed RBC. This is probably due to the fact that, in whole blood, an adsorption of plasma proteins on the NPs may occur and impact upon the haemolytic properties. This effect has already been reported in the literature for SiO_2_ NPs [32, 33]. Although various studies evaluated the haemolysis induced by Ag NPs [25, 34, 35], it remains difficult to compare results across studies due to the lack of harmonization of standardized physicochemical characterizations and *in vitro* haemolysis protocols. However, our results are in agreement with previous studies that concluded to the prohaemolytic properties of Ag NPs [25, 34, 35]. These results may have a clinical impact, since a release of haemoglobin can lead to adverse health effects such as anaemia, pulmonary hypertension, renal toxicity [36]. The mechanism explaining this induced haemolysis by Ag NPs has not yet been completely elucidated. It is known that low concentrations of Ag ions lead to RBC death [37, 38]. In contact with blood, metallic silver ionizes and releases ions in the bloodstream, which can interact with transmembrane proteins [2, 25]. However silver ion generation is not the only factor for haemolysis; other mechanisms contribute to haemolysis induced by NPs (deformability, adhesiveness, membrane vesiculation etc.) [25, 39].

Two techniques were used to assess the potential effect of Ag NPs on platelet function: LTA and FEG-SEM. With the first technique, Ag NPs did not affect the platelet aggregation at the highest tested concentration avoiding interferences (25 μg/mL). Jun *et al.* also showed interference of Ag NPs with light transmission, especially at high concentrations. With the second technique, an increase of the platelet adhesion is observed at 50 μg/mL, with no observed effect on the two following steps of the primary haemostasis (i.e., platelet activation and aggregation). Jun *et al.* and Krajewski *et al.* demonstrated that Ag NPs induce platelet activation and aggregation [26, 27]. An anti-aggregating effect of Ag NPs has been demonstrated by Shrivastava *et al.* using LTA [40]. However, the interference of Ag NPs was not taken into account with this technique. Moreover, as discussed by Deb *et al.*, the anti-platelet effect observed by Shrivastava *et al.* was due to citric acid present at the surface of the NPs [41]. The key mechanism underlying platelet activation induced by Ag NPs would be an increase of intracellular calcium levels, directly related to GPIIb/IIIa activation and leading to P-selectin expression and serotonin secretion [26].

On coagulation, the sensitive cTGT assay showed that Ag NPs had significant procoagulant activity. These results were in accordance with the work of Jun *et al.* which demonstrated the procoagulant activity of silver NPs <100 nm on two different assays (chromogenic generation assay and P-selectin expression) [26]. In the cTGT assays, the interferences (autofluorescence of the NP suspensions and the quenching of the fluorophore signal) are excluded by performing, for each NP concentration, a specific calibration curve run [22]. To explain the procoagulant effect observed, Jun *et al.* suggested that Ag NPs enhance procoagulant activity through phosphatidylserine exposure in platelets [26]. On the other side, Shrivastava *et al.* concluded to a delay of fibrin polymerization by silver NPs (10–15 nm). Interestingly, this effect is strongly inhibited when the experiment is performed with bovine serum albumin at a concentration below the protein concentration in the blood [28]. Martínez-Gutierrez *et al.* concluded to a moderate anticoagulant effect of Ag NPs on the intrinsic pathway performing an activated partial thromboplastin time (aPTT). We performed the same assay using a mechanical semi-automated coagulometer KC10 (Amelung, Germany) with Synthasil® (Instrumentation Laboratory, Lexington, KY, USA) and CK Prest® (Diagnostica Stago, Asnières, France). We observed a significant and dose-dependant decrease in coagulation with aPTT with Ag NPs (data not shown). Contrarily, we demonstrated a procoagulant effect of Ag NPs with cTGT mainly on the contact pathway. The conclusion of a procoagulant activity performed by cTGT should be regarded as more physiologically relevant and predictive. Indeed, reagents used in KC 10 contain, in addition to phospholipid substitutes, colloidal silica activator for Synthasil® and kaolin for CK Prest® to induce the contact pathway. cTGT reagents contain physiological phospholipids. In KC10, an interaction of charges between the negatively charged reagents and the Ag NPs delivering positive ions is anticipated. Consequently, this non-physiological interaction prevents tenase and prothrombinase complex formation, finally inducing an increase in the coagulation time. Moreover, procoagulant activity mediated by platelets could amplify the procoagulant effect observed on the coagulation cascade by Ag NPs. Because of the abovementioned interactions, aPTT using colloidal silica activator or kaolin to induce the contact pathway should not be considered as a relevant assay for assessing the impact of charged nanomaterials on coagulation. As suggested in previous work, cTGT appears to be the goldstandard assay [22].

Regarding biomedical applications, Ag NPs are currently used for wound dressing because of their antimicrobial effect [42]. Moreover, recent studies have shown that Ag NPs have significant anti-inflammatory effects [13, 43], preventing the wound from inflammation. In this study, we demonstrated a platelet proadhesion and a procoagulant effect promoting the healing process [44].

In the emerging field of nanomedicine, blood could be in contact with Ag NPs after injection or direct contact (coated biomaterial). We demonstrated prohaemolytic, proadherent and procoagulant effects of Ag NPs that could enhance the risk of thrombosis.

The most sensitive *in vitro* assay of the haemocompatibility testing battery for Ag NPs is the cTGT, used to propose a NOEC at 5 μg/mL and a LOEC at 50 μg/mL. The NOEC could be used to determine a safe human plasma-free concentration for a first-inhuman exposure and to identify appropriate safety margins. In addition, the LOEC could estimate the human plasma concentration where haematotoxicity would be expected to occur. As the human maximum therapeutic free plasma concentration would approach the toxic concentration, the probability of toxicity would increase and the exposure margin would decrease. The latter could also guide specific safety monitoring that would need to be foreseen in a clinical setting. As such, these *in vitro* hemocompatiblity data could contribute to the identification of starting and maximum doses as described in the ICH M3(R2) guideline on non-clinical safety studies for the conduct of human clinical trials and marketing authorization for pharmaceuticals [45]. It should be noted that the use of *in vitro* (haemato-) toxicity data in a decision-making process could be considered provided that the basic criteria of test method qualification are met [46].

## 5. Conclusion

Silver NPs present numerous interactions with blood components as RBCs, platelets and a coagulation system. We recommend specific haematological tests overcoming interferences to determine NP haemocompatibility and estimate NOEC. Using such tests, Ag NPs demonstrated prohaemolytic, procoagulant effects, and promote platelet adhesion. A maximal NOEC and LOEC in blood have been determined at 5 and 50 μg/mL, respectively. For local Ag NPs applications such as wound dressings, these properties may be favourable. For the development of Ag nanomedicines from a haemocompatibility perspective, the NOEC and LOEC may be useful for first-in-human exposure and for identifying appropriate safety margins.
